# Influenza

**Published:** 1892-01

**Authors:** Mathew Wilson

**Affiliations:** Mendota, Ill.


### INFLUENZA.*

By Mathew Wilson, M. R. C. V. S.

Influenza is a disease that has long been known to medical science, both human and veterinary. Its history can be traced back with certainty only to the beginning of the sixteenth century, although as far back as the year 1300 we have accounts of an epidemic among the horses of Italy that seems to-day to be recognized as influenza. With the beginning of the sixteenth century we have accounts of epidemics, the wide distribution of which have been reached by no other acute infectous disease.

Up to the present time a great number of epidemics have been described, which generally extended over whole countries and frequently over several quarters of the globe.

They returned at indefinite periods and affected every season and latitude, advancing, as a rule, in a great wave.

In some cases they appeared to be preceded by sporadic cases, but more commonly a large number of the animals would be affected simultaneously, the disease spreading with great rapidity.

Among the numerous outbreaks, the following are recorded:

In 1648 an epizootic of this disease appeared in Germany, and from there rapidly spread to other parts of Europe, and in 1711 it attacked the horses of the European armies, causing great losses.

In 1732 the disease appeared in London, and later on in the same century in Scotland.

In 1766 we have the first attack on the horses of America, but not making its appearance here in anything like a virulent form until we have the extensive outbreaks of 1870–72, when it spread over the entire country.

It is to-day an almost permanent disease among the horses of our large cities, where bad ventilation and want of sanitary arrangements about the great majority of stables seem to keep the disease alive, and perhaps predispose fresh animals to it.

Definition.—Influenza is a specific febrile disease, dependent upon a specific blood poison and prevailing as an epizootic.

It is essentially characterized by a catarrh of the respiratory and generally also of the digestive organs, by great and rapidly developed weakness, pains in the head and limbs, as well as by

* Read before the Illinois Veterinary Medical Association.

serious nervous symptoms and fever of greater or less intensity.

It is confounded generally with simple catarrh, but is distinguished by its wide diffusion, its rapid spread and the number of cases in the regions in which it occurs.

We cannot lay its cause to atmospheric influences, as we have it occurring at all times of the year, during different climatic changes and in countries whose atmospheric surroundings are totally different.

We have it occurring at seasons of the year when climatic changes are such as do not produce catarrh, and aside from this we have those lesions of function peculiar to influenza, that can in no way be connected with a simple catarrh.

Etiology.—When we think of the numerous opportunities presented by this disease for investigation, and to what extent literature has been published upon it, we are surprised at what few facts have been gathered together concerning its cause and origin.

A great many theories have been advanced as to the etiology of influenza, such as that of atmospheric influence; others give it a specific origin, but have never been able to isolate and demonstrate its specific cause, while on the other hand there are those who claim it has a spontaniety of origin, due to want of sanitation. This last is, I think, the weakest of all, as we have it occurring where sanitary arrangements are the best, as well as where they are almost entirely wanting.

The theory of its specific origin is, I think, conceded by the majority to be the correct one, although we have as yet been unable to produce conclusive evidence.

It is due to a *living miasm*, capable of being carried onward by the air, but having an independent existence of its own, and which would find in certain places conditions more favorable for its development than in others.

Take, for example, the last outbreaks of influenza in the human family, which seemed to have been developed in Russia and spread in the direction of human intercourse and the prevailing winds from the east to the west.

This living miasm is capable of transmission through the air, of being carried by human beings, or, in fact, by any of the known modes of infection.

Influenza has been described as the sum of a series of catarrhal manifestations, which have developed under common epidemic influences, and the intimate association of the various local affections allows us to give them a common specific origin.

Many acute local affections, such as acute catarrh, laryngitis, etc., present very much the same symptoms, locally, as in this disease, but there is wanting the sudden and general seizure, the severe nervous depression, and the extent to which the mucous membranes are involved—all these seem in favor of a general cause which has a specific effect upon the whole body.

These symptoms are much more severe than in the local affections, while they remind us more of analagous symptoms in other acute infectious diseases, and for these reasons I think we are justified in classing it under the same group.

There is a close analogy between the first symptoms of influenza and measles in the human subject.

Before the eruption occurs on the skin in measles, there is found to be a catarrhal affection of the mucous membranes lining the air passages, and also of the conjunctiva. This catarrh is so constant a manifestation that it has been considered a pathognomic symptom, especially in those cases where the eruption cannot be seen. Here it is, as in influenza, one of the earliest and most constant symptoms.

In canine distemper we have another disease, whose early symptoms coincide with those of influenza.

Here we have the disease ushered in with chills, a dry, irritated condition of the mucous membranes, where the discharge soon becomes more copious, great debility, and in some cases an extension of the inflammation along the respiratory tract to the lungs and pleura.

In these diseases we have two that are recognized as being due to a specific organism, presenting characteristic symptoms that almost coincide with those of influenza; and what more probable to assume from this, that in influenza we also have a disease whose ravages are due to a similar cause.

Pathology.—The pathological changes in the body are due to the absorption of the morbid material by the blood. The alteration occurs in the blood, where we have a rapid destruction of the red corpuscles. The absorption by the tissues of these disintegrated corpuscles give them a yellowish tint and a congested appearance. The first sign of this is seen in the early discoloration of the mucous membranes.

Along with this we always have more or less congestion of the various organs of the body.

Other pathological changes are due to complications; as, if the lungs are affected, we have the changes due to pneumonia or pleurisy. If enteritis or congestion of the liver is the complication, we have the changes taking place in them.

Symptoms.—The development of the symptoms of this disease, after a period of incubation varying from four to eight days, may result in a very mild attack, or they may be very intense.

In a mild attack we have the disease running its course as a specific fever, with only the alterations in the blood; but if the attack is severe, we may have it complicated with inflammatory diseases of the various organs, aggravated by the already weakened state of the body and the alterations in the blood, which have a tendency to favor a fatal termination of these complications.

The first symptoms are those of great indisposition, rapidly developing fever, which may become intense, chills of the body, staring coat, loss of appetite, and a dry, irritated condition of the mucous membranes.

The pulse becomes increased in number, varying from 60 to 80 and even 100; it may be at first moderate in volume, but becomes weak.

The discharge from the mucous membranes at first is thin and acrid, but as the disease advances it becomes more copious and thicker.

In the condition that is known as pink eye we have the discolored pink condition of the mucous membranes lining the nasal and buccal cavities and the eyelids, tumefaction of the limbs and eyelids, great stupor, and the animal very weak.

The fever may run up as high as 105 degrees F. or 106 degrees F., and generally lasts from three to four days.

At the end of this time, if the disease runs a favorable course, the fever begins to abate, the appetite returns, the various organs take on their natural function, the pulse falls in number and becomes stronger, and we have the animal left convalescent in a weakened condition.

Death in these cases may be the result of an excessive fever, with failure of the heart's action, asphyxia from a rapid congestion of the lungs, or from the poisonous effect of the morbid matter due to disintegration of the blood corpuscles.

Complications—The complications, as we have before mentioned, are generally of an inflammatory nature. As a result of the primary lesion, we have a congestion of the various tissues.

This, along with a distended state of the blood vessels, a weak heart's action and an improper aeration of the blood, is very prone to be followed by an inflammation, due to the slightest irritating cause.

During some outbreaks we have the majority of cases complicated with an inflammatory condition of the lungs; in others we have the complications arising in the bowels or liver. Why this should be we cannot determine, unless it is that local climatic changes or atmospheric influences may be the exciting cause of these local lesions, the animal becoming more predisposed, due to the pre-existing disease.

To enumerate the symptoms of the various complications would be to go into those of pneumonia. pleurisy, enteritis, etc., which I do not think would throw any light on our subject, and which could only be thoroughly discussed under their respective heads.

Treatment.—Treatment must, of course, depend upon the symptoms exhibited by each particular case; but there are some measures that will equally apply to all.

Great care must be taken to keep the animal free from exposure to draughts, and at the same time have ventilation sufficient to provide him with plenty of fresh air.

He should be well covered with sufficient blankets to keep up external heat, the legs hand rubbed and bandaged, and his surroundings kept clean.

Antipyrities are indicated from the first. Of these we have a great variety, and selection must depend upon the practitioner.

I have found a comhination of digitalis and nitrate of potash a good remedy, giving it twice a day.

In this we have not only a febrifuge action, but we strengthen the heart, lower its pulsations and have a diuretic effect.

If the fever remains high, two or three doses of acetanilid, combined with digitalis, sometimes has a good effect.

If the attack is mild, generally, all that is needed is good nursing and salines dissolved in the drinking water.

If there is a tendency to constipation, a powder of sulphur and nitrate of potash each day will generally relieve it, along with warm bran drinks or linseed tea.

The treatment in complications must, of course, depend upon the accompanying disease, remembering at the same time the weakened state of the animal, and let our treatment be such as will keep up our patient's strength.

Mendota, Ill.

